# Influence of Hesperidin on the Physico-Chemical, Microbiological and Sensory Characteristics of Frozen Yogurt †

**DOI:** 10.3390/foods13050808

**Published:** 2024-03-06

**Authors:** Roberto Cedillos, Ricardo S. Aleman, Ryan Page, Douglas W. Olson, Charles Boeneke, Witoon Prinyawiwatkul, Kayanush Aryana

**Affiliations:** School of Nutrition and Food Sciences, Louisiana State University Agricultural Center, Baton Rouge, LA 70803, USA; rcedil1@lsu.edu (R.C.); rsantosaleman@lsu.edu (R.S.A.); rpage1@lsu.edu (R.P.); douglas.olson2@usda.gov (D.W.O.); cboene1@lsu.edu (C.B.); wprinya@lsu.edu (W.P.)

**Keywords:** probiotics, hesperidin, polyphenols, frozen yogurt, sensory

## Abstract

Frozen yogurts contain yogurt culture bacteria, which might impart health benefits to their consumers. Global frozen yogurt market sales are expected to grow by 4.8% by 2028, which represents an important opportunity for the industry, consumers and researchers. Polyphenols are metabolites found in plants which have antioxidant and anti-inflammatory properties and might prevent chronic diseases such as cancer, diabetes and cardiovascular diseases. The objective of this study was to elucidate the effect of the polyphenol hesperidin on the physico-chemical, microbiological and sensory characteristics of frozen yogurts. Hesperidin was incorporated into frozen yogurt at three concentrations (125, 250 and 500 mg/90 g of product), while yogurt with no hesperidin was used as a control. The viscosity and overrun of the frozen yogurt were analyzed on day 0. The hardness, pH, color and *Lactobacillus bulgaricus* and *Streptococcus thermophilus* counts were determined after 0, 30 and 60 d. The melting rate was determined at 60 and 90 min after 0, 30 and 60 d. The bile and acid tolerances of both *S. thermophilus* and *L. bulgaricus* were measured after 7 and 60 d. A hedonic scale of nine points was used to measure sensory attributes. Data were analyzed at α = 0.05 with an ANOVA with Tukey’s adjustment, and McNemar’s test was used to analyze purchase intent. Hesperidin did not influence the pH, overrun or microbial characteristics. Polyphenol addition compared to the control decreased the melting rate but increased the hardness and bile tolerance of *L. bulgaricus*, as well as the L* and b* values. The sensory characteristics were not influenced by the lowest concentration of hesperidin, as it was not statistically different from the control. Moreover, consumers were interested in purchasing frozen yogurt with added hesperidin after learning about the health claim. This study can assist in the development of a healthier frozen yogurt in an increasingly competitive market.

## 1. Introduction

Milk-based frozen desserts are very popular in the human diet. These types of desserts have had a very important role in society since there are records of their consumption dating back to the 11th century [1]. Among them, frozen yogurt is a dairy-based product that is popular among consumers. This product is characterized by an acidic taste that has the refreshing and cold features of an ice cream. It can be served soft, hard or in a mousse way [2].

One of the most important benefits of yogurt (including frozen yogurt) is its probiotic bacteria content since US yogurt regulation requires it to be produced by culturing *Lactobacillus bulgaricus* and *Streptococcus thermophilus,* as well as potentially other bacteria, containing at least 10^6^ CFU/g during its shelf life [3]. It is important to highlight that if the product is heat treated after fermentation, the bacteria are killed [4]. Probiotic bacteria are defined as “living microorganisms that, when consumed, have the potential to confer a beneficial health effect” [5]. At the same time, the FAO (Food and Agricultural Organization of the United Nations) and the WHO (World Health Organization) have defined probiotics as “live strains of strictly selected microorganisms which, when administered in adequate amounts, confer a health benefit on the host” [6]. Safety, functionality and technological usability are some important characteristics that bacteria should fulfill. Yogurt consumption has been shown to be associated with a better overall diet quality and a healthier metabolic profile [7].

Polyphenols comprise one of the most numerous and widely distributed group of substances in the plant kingdom and are mainly found in fruits and vegetables. These compounds are the secondary plant metabolites that carry one or more hydroxyl groups [8]. Food polyphenols are categorized according to their structure as phenolic acids, flavonoids, stilbenes and lignans [9]. Moreover, these molecules have antioxidant properties and the ability to regulate enzymatic activities [10]. On the other hand, reactive oxygen species are defined as molecules which have oxygen in their structure, may have a charge (either positive or negative), and possess the ability to oxidize other molecules [11]. Related to this, oxidative stress is understood as an imbalance that exists between oxidizing agents such as reactive oxygen species (ROS) and antioxidants such as superoxide dismutase (SOD) or polyphenols [12]. Hesperidin is defined as “a flavanone glycoside consisting of the flavone hesperitin bound to the disaccharide rutinose” [13], and this compound is the major polyphenol (flavonoid) found in citrus fruits such as lemons and oranges, containing up to 41 and 60 mg per 100 mL of juice, respectively [14]. It can also be found in other types of fruits. This compound is antihypertensive, antidiabetic and cardioprotective due to its antioxidant action by reducing the production of inflammatory markers such as cytokines [15]. Moreover, this polyphenol has demonstrated antimicrobial activity against pathogenic bacteria [16]. An important characteristic of this polyphenol is its stability up to 80 °C for 10 min, meaning that it is heat-stable for most pasteurization treatments [17]. Hesperidin is stable from a pH of 1 to 7 [18].

There has been no research that investigates the effects of incorporating hesperidin into frozen yogurt. As a result, this study aimed to determine the effect of added hesperidin on the physicochemical, microbial and sensory characteristics of frozen yogurt. Also, the bile and acid tolerances of *L. bulgaricus* and *S. thermophilus* in frozen yogurt were evaluated.

## 2. Materials and Methods

### 2.1. Experimental Design

The polyphenol hesperidin at different concentrations constituted the treatments. Three concentrations of hesperidin were incorporated into frozen yogurt at 125, 250 and 500 mg per 90 g serving, while the negative control did not contain hesperidin (Table 1). All experiments were replicated three times.

Various experimental designs were used. First, the viscosity and overrun of the frozen yogurt mix on day 0 were measured using a randomized block design where, the blocks were the replications. *S. thermophilus* counts, *L. bulgaricus* counts, pH, hardness and color were analyzed with two factors (hesperidin concentrations and days of storage (0, 30 and 60 d)) in a randomized block design, where the replications were the blocks. A factorial arrangement with three factors (hesperidin concentration, days of storage and minute/hour) in a randomized block design was analyzed at a melting rate of 60 and 90 min on days 0, 30 and 60, and the bile tolerances of *S. thermophilus* and *L. bulgaricus* (0, 4 and 8 h) and the acid tolerances of *S. thermophilus* (0, 1 and 2 h) and *L. bulgaricus* (0, 15 and 30 min) after 7 and 60 d were measured. The sensory attributes of the frozen yogurt on day 60 were analyzed as a completely randomized design using 103 consumers, where each consumer was a replication.

### 2.2. Frozen Yogurt Production

The frozen yogurt was produced at Louisiana State University, as described in Table 1 [19]. After preheating locally purchased pasteurized whole milk within 8 L stainless steel containers to 70 °C ± 2 °C, the appropriate amounts of sugar (Domino Foods, Inc., Yonkers, NY, USA), maltodextrin (Bulk Supplements, Henderson, NV, USA), grade A nonfat dry milk (Dairy America, Fresno, CA, USA), locally purchased corn starch (Argo, Oakbrook Terrace, IL, USA) and hesperidin purchased online (Bulk Supplements, Henderson, NV, USA) were added according to each formulation and mixed with stainless steel stirring rods until all the ingredients were properly incorporated. The mixes were then pasteurized at 75 ± 1 °C for 30 min, tempered to 43 ± 1 °C, inoculated with a 1:1 ratio of *L. bulgaricus* (LB-12) and *S. thermophilus* (ST-06) (Chr. Hansen’s Laboratory, Milwaukee, WI, USA) and fermented in an incubator (model 815 Thermo Scientific, Two Rivers, WI, USA) at 43 ± 1 °C until the pH reached 4.7 ± 0.1. Yogurts were then stored at 4 ± 1 °C for 24 h of aging. The mixes were manually stirred for 60 s to break down the coagulum until no clumping was observed and then poured into a batch freezer (Emery Thompson 20 NW, Brooksville, FL, USA) for freezing for 9 min 30 s. Finally, the frozen yogurts were packed into 59, 163, 355, 473 and 946 mL polypropylene containers with lids and stored at −25 ± 2 °C until analyses at various storage times (0, 30 and 60 days).

### 2.3. Frozen Yogurt Physicochemical Characteristics

#### 2.3.1. Overrun

The overrun was measured according to Muse and Hartel [20]. The overrun percentage (%) was calculated by subtracting the weight of a given volume of frozen yogurt after freezing from the weight of the same volume of frozen yogurt before freezing, then dividing this difference by the weight of a given volume of frozen yogurt after freezing it and expressing this quotient as a percentage.

#### 2.3.2. Viscosity

The viscosity of the frozen yogurt mix was determined according to Aryana [21], with slight modifications. On d 0, yogurt mixes were stirred manually for 60 s at 4 ± 1 °C in 946 mL containers (13.97 cm height, 11.43 cm top diameter and 8.89 cm bottom diameter) (Pantry Value, Converse, TX, USA) to measure the viscosity. A Brookfield viscometer model SV-22 (Brookfield Engineering Lab Inc., Middleboro, MA, USA) incorporating the Wingather^®^ 32 software (Brookfield Engineering Lab Inc.) and an RV 5 spindle rotating at 20 rpm were used to obtain the viscosity. An average of 20 data points was used for the analysis.

#### 2.3.3. Hardness 

Before the hardness of the frozen yogurt was measured, the samples were tempered to −18 ± 1 °C for 24 h to simulate the hardness of the product at a consumption temperature in 355 mL cups (6.35 cm height, 11.43 cm top diameter and 8.89 cm bottom diameter). A texture analyzer (TA.XT Plus Connect, Texture Technology Corp., Hamilton, MA, USA) with a 50 kg load cell and a TA-43R probe was used with a 2.0 mm/s pre-test speed, a 3 mm/s test speed and a 10.0 mm/s post-test speed. The hardness was measured as the peak force (N) needed to penetrate the product by 3.5 cm at the geometrical center of the container. The measurements for hardness were performed on days 0, 30 and 60.

#### 2.3.4. pH

The samples used for pH measurements were completely melted and then analyzed at 25 ± 1 °C using an Orion Star™ A111 (Thermo Fischer Scientific Inc., Waltham, MA, USA) pH meter. The measurements for pH were performed on days 0, 30 and 60. Before its use, the instrument was calibrated using reference pH 4 and 7 buffer solutions. 

#### 2.3.5. Color

Color was measured using a MiniScan XE Plus portable handheld color spectrophotometer (HunterLab, Reston, VA, USA). The instrument was calibrated with black and white standard reference tiles. L*, a* and b* values were taken as the average of three consecutive measurements per replication. The color measurements were performed on days 0, 30 and 60. 

#### 2.3.6. Melting Rate

The melting rate was measured according to Januário et al. [22], with slight modifications. When frozen yogurts were manufactured, 90 g samples of semi-soft consistency were placed into 163 mL containers (6.03 cm height, 7.30 cm top diameter and 4.76 cm bottom diameter). The frozen yogurt samples in containers were tempered at 18 ± 1 °C for 24 h prior to testing. The samples were removed from their containers and placed over a 1-mesh stainless steel net within a funnel over a graduated cylinder in order to melt at 25 ± 1 °C. The volume of melted product was recorded after 60 and 90 min. The measurements were performed on days 0, 30 and 60.

### 2.4. Frozen Yogurt Microbial Characteristics

#### 2.4.1. Enumeration of *S. thermophilus*

An *S. thermophilus* agar was prepared according to Dave and Shah [23]. Sucrose (10 g) (Amresco, Solon, OH, USA), yeast extract (5 g) (Becton Dickinson and CO., Sparks, MD, USA), dipotassium phosphate (K_2_HPO_4_) (2 g) (Fischer Scientific, Fair Lawn, NJ, USA) and bacto tryptone (10 g) (Becton Dickinson and Co., Sparks, MD, USA) were dissolved in 1 L of distilled water. The pH was adjusted to 6.8 ± 0.1 with 1 *N* of HCl before adding 12 g of agar and 30 mg of bromocresol purple (Fisher Scientific, Fair Lawn, NJ, USA) and autoclaving at 121 °C for 15 min. Samples were serially diluted using 0.1% peptone solution (Becton Dickinson and Co., Sparks, MD, USA), pour-plated and incubated aerobically for 24 h at 37 ± 1 °C. The colony-forming units were counted using a colony counter (Quebec Darkfield, Leica Inc., Buffalo, NY, USA). The enumeration of *S. thermophilus* was performed after 0, 30 and 60 d in triplicate.

#### 2.4.2. Enumeration of *L. bulgaricus*

An MRS agar was prepared to enumerate *L. bulgaricus*. Lactobacilli MRS broth (55 g) (Becton Dickinson and Co., Sparks, MD, USA) and 15 g of agar (Fisher Scientific, Fair Lawn, NJ, USA) were mixed with 1 L of distilled water. The pH was adjusted to 5.2 ± 0.1 using 1 *N* of HCl before autoclaving at 121 °C for 15 min. Samples were serially diluted using 0.1% peptone solution (Becton Dickinson and Co., Sparks, MD, USA), pour-plated and incubated aerobically for 72 h at 43 ± 1 °C. The colony-forming units were counted using a colony counter (Quebec Darkfield, Leica Inc., Buffalo, NY, USA). The enumeration of *L. bulgaricus* was performed after 0, 30 and 60 d in triplicate.

#### 2.4.3. Bile Tolerance of *S. thermophilus* and *L. bulgaricus*

The preparation of the broths and procedures was performed according to Pereira and Gibson [24], with some modifications. For the determination of the bile tolerance of *L. bulgaricus*, an MRS-THIO broth was prepared by mixing 55 g of MRS lactobacilli broth (Becton Dickinson and Co., Sparks, MD, USA) per liter of distilled water and supplementing with 0.3% of oxgall (bile salts) (US Biological, Swampscott, MA, USA) and 0.2% sodium thioglycolate (Acros Organics, Fair Lawn, NJ, USA) as an oxygen scavenger. The broth was then autoclaved at 121 °C for 15 min, tempered to 43 ± 1 °C and supplemented with 0.5% lactose. The MRS-THIO broth (99 mL) was inoculated with *L. bulgaricus* (11 mL). The procedures used for preparing dilutions, pour plating, incubating and counting the colonies of *L. bulgaricus*, as previously described, were the same as previously described. The bile tolerance of *L. bulgaricus* was measured on days 7 and 60. The enumeration was carried out in triplicate.

For the determination of the bile tolerance of *S. thermophilus*, an M17 broth (37.25 g) (Becton Dickinson and Co., Sparks, MD, USA) was mixed with 950 mL of distilled water and supplemented with 0.3% oxgall (bile salts) (US Biological, Swampscott, MA, USA). This broth was autoclaved at 121 °C for 15 min, tempered to 37 ± 1 °C and supplemented with 0.5% lactose. Next, 11 g of *S. thermophilus* was inoculated into this M17 oxgall broth (99 mL). Although an M17 agar was used instead of an *S. thermophilus* agar for pour plating, the procedures used for preparing the dilutions, pour plating, incubating and counting the colonies of *S. thermophilus* were the same as previously described. This enumeration was carried out using the same diluted sample in the M17 broth and enumerated after 0, 4 and 8 h. The bile tolerance of *S. thermophilus* was measured on days 7 and 60 in triplicate.

#### 2.4.4. Acid Tolerance of *S. thermophilus* and *L. bulgaricus*

The preparation of the broths and procedures was carried out following the method used by Pereira and Gibson [24], with some modifications. For the determination of the acid tolerance of *L. bulgaricus*, 55 g of a lactobacilli MRS broth (Becton Dickinson and Co., Sparks, MD, USA) was mixed with 1 L of distilled water, and the MRS broth was acidified using HCl. The broth was autoclaved at 121 °C for 15 min and tempered to 43 ± 1 °C until use. Eleven grams of the sample was tenfold diluted into the MRS broth acidified previously prepared and serially diluted using 0.1% peptone water (Becton Dickinson and CO., Sparks, MD, USA). The acidified MRS broth (99 mL) was inoculated with *L. bulgaricus* (11 mL). The procedures used for preparing the dilutions, pour plating, incubating and counting the colonies of *L. bulgaricus*, as previously described, were the same as previously described. This enumeration was performed after 0, 15 and 30 min on days 7 and 60 in triplicate.

For the determination of the acid tolerance of *S. thermophilus*, 37.25 g of M17 broth powder (Becton Dickinson and Co., Sparks, MD, USA) was mixed with 950 mL of distilled water. This M17 broth was acidified using HCl, autoclaved at 121 °C for 15 min, tempered to 37 ± 1 °C and supplemented with 0.5% lactose. The acidified M17 broth (99 mL) was inoculated with *S. thermophilus* (11 mL). Although an M17 agar was used instead of an *S. thermophilus* agar for pour plating, the procedures used for preparing the dilutions, pour plating, incubating and counting the colonies of *S. thermophilus* were the same as previously described. This enumeration was performed after 0, 1 and 2 h on d 7 and 60 in duplicate per repetition.

### 2.5. Sensory Analysis of Frozen Yogurt

The sensory study was exempted from oversight by the LSU Institutional Review Board with the IRB exception number IRBAG-22-0076. The 103 random participants in this study accepted informed consent where the potential risks were explained. The selection criteria required participants to be 18 years of age or older and have no adverse reactions to milk products. Males represented 49% and females 51%. Moreover, the age distribution was dominated by 18–25 year olds (76%), 26–35 year olds (16%) and the remaining 8% were people above 35 years old. The racial conformation was led by white/Caucasian, with 43%, followed by Latino, black or African American and Asian, with 18, 18 and 14%, respectively. Finally, 7% identified themselves as other races. All participants were given four treatments of frozen yogurt with three different concentrations of hesperidin (125, 250 and 500 mg per 90 g serving) along with a negative control, served in 59 mL polypropylene containers with lids. A 9-point hedonic scale was used in this study, where 9 meant like extremely, 1 meant dislike extremely and 5 meant neither like nor dislike. Appearance, color, aroma, texture, iciness/sandiness, flavor, sourness and overall liking were evaluated. Additionally, purchase intent was asked before and after a health statement regarding the polyphenol presence in the provided product was given.

### 2.6. Statistical Analysis

All data were analyzed using a Statistical Analysis System (SAS^®^ version 9.4) using either PROC GLM or PROC ANOVA. The means and standard deviations were reported in triplicate. The viscosity and overrun of the frozen yogurt were analyzed in a randomized block design on day 0. The hardness, pH, color and *L. bulgaricus* and *S. thermophilus* counts were analyzed as a 2-factor factorial arrangement within a randomized block design. A factorial arrangement with three factors (hesperidin concentration, minutes/hours and day) in a randomized block design was used for the analysis of the melting rate, the bile tolerances of both *S. thermophilus* and *L. bulgaricus* and the acid tolerances of both *S. thermophilus* and *L. bulgaricus*. Sensory data were analyzed in a completely randomized design. For physicochemical and microbial properties, PROC GLM was used with Tukey’s adjustment. The differences of least squares means were used to determine the statistical differences (*p* ˂ 0.05) for the main effects (treatments, storage time (day) and hour/minute) and their interactions. For sensory attributes, an ANOVA was performed with Tukey’s adjustment. Significant differences in purchase intent before and after a health claim were determined using a McNemar’s test.

## 3. Results and Discussion

### 3.1. Viscosity of Yogurt Mix

**Figure 1:**
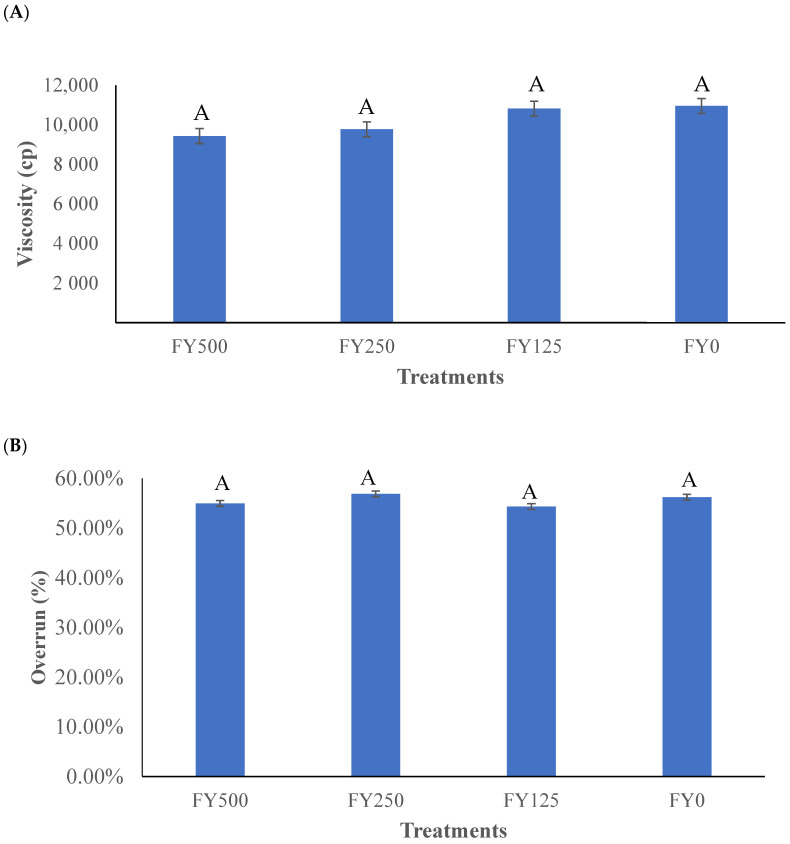
(**A**) Viscosity of the frozen yogurt mixes with hesperidin added before freezing in the batch freezer. (**B**) Overrun measurements in frozen yogurt with added hesperidin. FY500 = frozen yogurt with 500 mg of hesperidin per 90 g serving. FY250 = frozen yogurt with 250 mg of hesperidin per 90 g serving. FY125 = frozen yogurt with 125 mg of hesperidin per 90 g serving. FY0 = frozen yogurt without added hesperidin. ^A^ bars not containing a common letter are significantly (*p* < 0.05) different.

The viscosities of the frozen yogurt mixes on day 0 are presented in Figure 1A. No statistical (*p* > 0.05) differences were found between the treatments. An explanation of these results might be the standardized process of 60 s of stirring to break the coagulum that formed in the fermentation steps that all frozen yogurt mixes experienced before being poured into a batch freezer. Another reason could be due to the low concentrations of added hesperidin, which ranged from 14.85 to 59.66 g. Consequently, the hesperidin addition did not affect the viscosities of the frozen yogurt mixes when compared to a plain frozen yogurt mix.

Viscosity is important for quality assurance purposes and is defined as the resistance to flow of a certain substance. Low-viscosity substances will easily deform and modify their shape [25]. Fenelon et al. [26] established that yogurt viscosity is related to the amount of protein and fat and the proportions of casein and whey protein, which is in agreement with the results found in this study.

### 3.2. Overrun of Frozen Yogurt

Overrun is an important property for frozen dairy desserts and represents the percentage of air that a product contains in its matrix. This property might influence other characteristics such as the hardness, melting rate and mouthfeel [27].

The overrun results for the frozen yogurts as influenced by the hesperidin concentration are shown in Figure 1B. No statistical (*p* > 0.05) differences were found among the treatments. These results can be explained by the standardized procedure of freezing the frozen yogurt for 9 min and 30 in the batch freezer during manufacturing. Another possible factor for the lack of a significant difference is that hesperidin has poor water solubility [28]. The foaming capacity of hesperidin is limited since a good foaming agent, like proteins, should decrease the surface tension between air and water [29,30]. In other words, the hesperidin addition did not affect the overrun capacity.

### 3.3. Microbial Analysis of Frozen Yogurt

**Figure 2:**
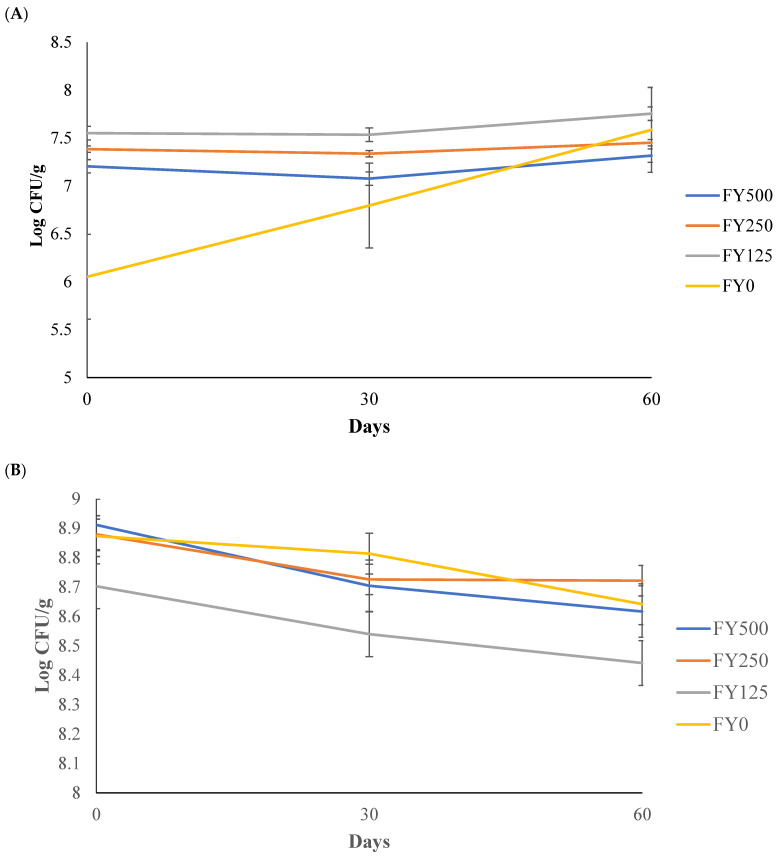
(**A**) *L. bulgaricus* counts in frozen yogurt with added hesperidin over 60 d of storage. (**B**) *S. thermophilus* counts in frozen yogurt with added hesperidin over 60 d of storage. FY500 = frozen yogurt with 500 mg of hesperidin per 90 g serving. FY250 = frozen yogurt with 250 mg of hesperidin per 90 g serving. FY125 = frozen yogurt with 125 mg of hesperidin per 90 g serving. FY0 = frozen yogurt without added hesperidin.

The *L. bulgaricus* log_10_ counts in frozen yogurt over 60 d of storage are illustrated in Figure 2A. The main effects (ingredient and day) and the interaction effect (ingredient × day) were not significant (*p* > 0.05). This stability for *L. bulgaricus* counts in a frozen environment was also found by Davidson et al. [31], where the survival of *L. bulgaricus* in frozen yogurt showed no statistical differences over 11 wk of storage. Also, Alfaro et al. [19] found that the probiotic bacteria count in frozen yogurt containing purple rice bran oil remained stable throughout 6 wk of storage. Furthermore, Olson et al. [32] did not find decreases in *L. bulgaricus* counts after 8 wk of storage in yogurt ice cream.

The *S. thermophilus* log_10_ counts in frozen yogurt over 60 d of storage are presented in Figure 2B. The main effects (ingredient and day) were significant (*p* < 0.05), whereas the interaction effect (ingredient × day) was not significant (*p* > 0.05). The lowest *S. thermophilus* counts were found in treatment FY125, whereas the other treatments did not differ from the control samples. Furthermore, the storage time had a significant (*p* < 0.05) effect on the *S. thermophilus* counts, where reductions were observed on days 30 and 60 when compared to day 0. Even though statistical (*p* ˂ 0.05) differences were found in both cases, the reductions were less than half of one log CFU/g, which can be considered practically no difference between treatments and days. These results are similar to those found by Atallah et al. [33], where they discovered that frozen yogurt with different types of sweeteners reduced the *S. thermophilus* counts during 60 days of storage. The freezing process might injure bacteria through mechanical damage to their cell walls, increased solutes in the extracellular medium and dehydration [34], which may explain the slight reductions seen in this study.

For both bacteria, the lactic acid bacteria (LAB) counts remained stable throughout the storage period, showing that the presence of hesperidin did not negatively impact the bacteria counts throughout storage. Both bacteria had counts of at least 10^6^ CFU/g, which is the concentration needed in probiotic foods [35] and what is required by US regulations [3].

### 3.4. The pH of Frozen Yogurt

**Figure 3:**
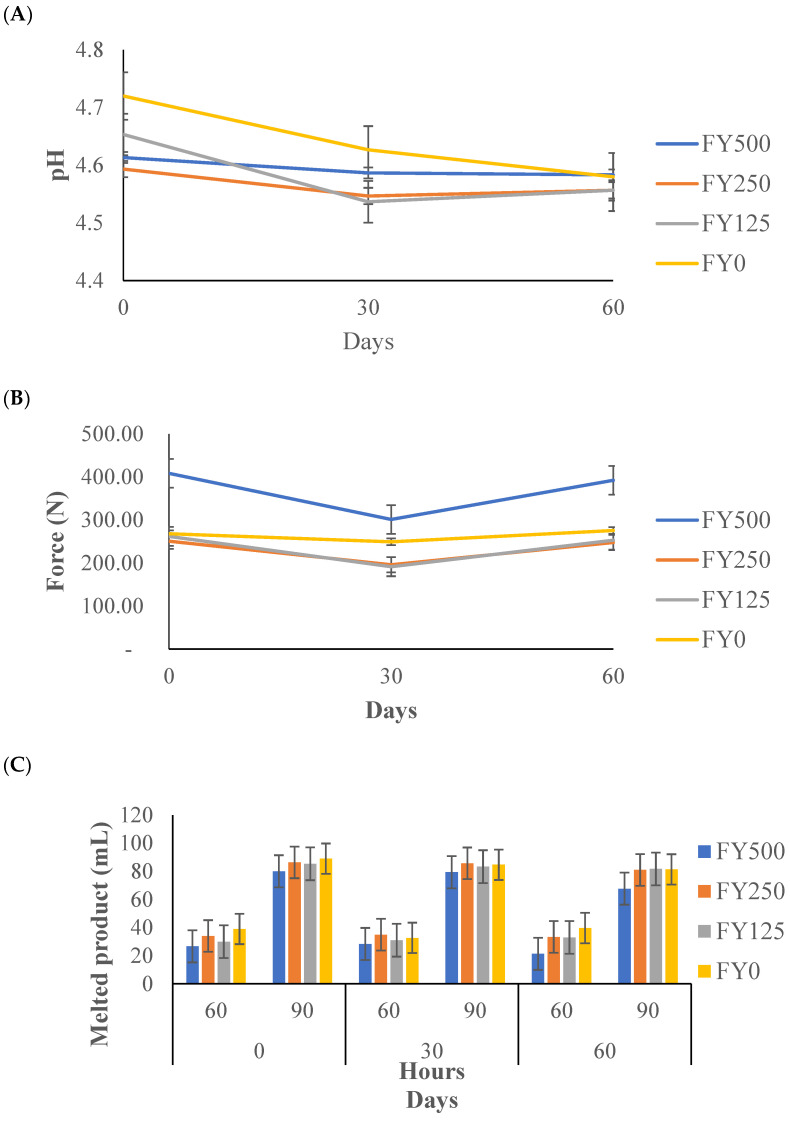
The pH (**A**), hardness (**B**) and melting rate (**C**) measurements of frozen yogurt with added hesperidin over 60 d of storage. FY500 = frozen yogurt with 500 mg of hesperidin per 90 g serving. FY250 = frozen yogurt with 250 mg of hesperidin per 90 g serving. FY125 = frozen yogurt with 125 mg of hesperidin per 90 g serving. FY0 = frozen yogurt without added hesperidin.

The pH results for the frozen yogurt are shown in Figure 3A. The main effects (ingredient and day) and the interaction effect (ingredient × day) were not significant (*p* > 0.05). The values ranged from 4.54 to 4.72, showing relative pH stability. Two factors might explain the lack of significance (*p* ˂ 0.05) for pH. First, the controlled endpoint of the fermentation during the frozen yogurt manufacturing process was continued until reaching a pH of 4.7 ± 0.1. Second, chemical and microbial reactions are stable in a freezing environment [36]. These results are very similar to the results found by Inoue et al. [37] for stable pH values for frozen yogurt kept during six months of storage.

The pH is a measurement of the acidity or alkalinity of a product in an aqueous solution and is normally between 0 and 14 [38]. In the dairy industry, pH measurement is a very important parameter since acidic components directly affect the stability, flavor and shelf life of dairy products [39].

### 3.5. Hardness of Frozen Yogurt

The hardness results are illustrated in Figure 3B. The treatment effect was significant (*p* < 0.05), but the day effect and the interaction effect (ingredient × day) were not significant (*p* > 0.05). The frozen yogurt containing the highest hesperidin concentration recorded the highest force for hardness (in Newtons), whereas the hardness of the other treatments did not differ from the hardness of the control samples. This increased hardness of the frozen yogurt containing the highest hesperidin concentration could be related to its higher total solids content [40]. Ghelich et al. [41] demonstrated that the inclusion of wheat germ protein hydrolysates at 0.5, 1 and 1.5% concentrations significantly increased the hardness of frozen yogurt. Factors such as ice crystal sizes, fat content, overrun and solid content affect the melting rate [20].

### 3.6. Melting Rate of Frozen Yogurt

The melting rate results are presented in Figure 3C. The treatment effect and day effect were significant (*p* < 0.05), while all the other effects were not significant (*p* > 0.05). The treatment that had the lowest melting rate was the FY500, with only 50.5 mL melted at 60 d of storage, whereas the melting rate of the other treatments did not differ from the melting rate of the control samples. As expected, the frozen yogurt continually melted over time. 

The melting rate is a very important characteristic for frozen dairy desserts and is defined as the resistance to melting. It is influenced by the fat and stabilizer content, the initial product temperature, the ambient temperature and air cells [42]. Muse and Hartel [20] established that the main components that influence melting rate are fat destabilization, ice crystal size, overrun and rheological properties. Moreover, Li et al. [43] demonstrated that polyphenol–protein complexes have the potential to improve the heat stability (lower the melting rate) of a sample. The results obtained in this study are like those found by Gabbi et al. [44], where they tested the melting rate properties of ice cream supplemented with 0.50 to 2.0% ginger powder rich in polyphenols and found that the melting rates of the treatments were different from the melting rate of the control ice cream. Also, Bilbao et al. [45] added strawberry powder to a dairy frozen dessert to enhance its heat stability.

### 3.7. Color of Frozen Yogurt

**Figure 4:**
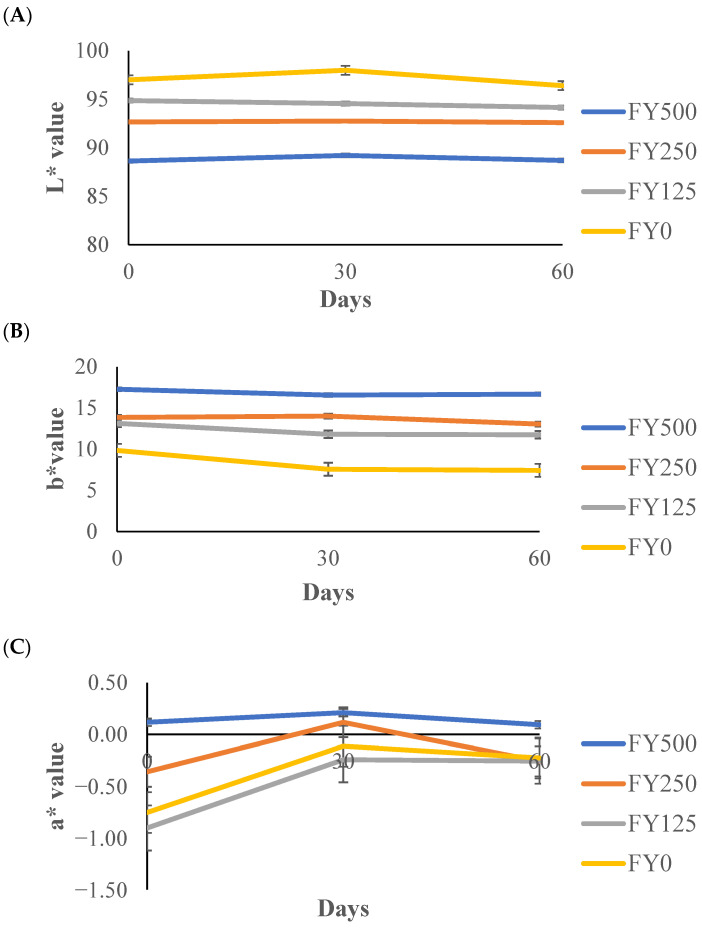
The L* (**A**), b* (**B**) and a* (**C**) values of frozen yogurt with added hesperidin over 60 d of storage. FY500 = frozen yogurt with 500 mg of hesperidin per 90 g serving. FY250 = frozen yogurt with 250 mg of hesperidin per 90 g serving. FY125 = frozen yogurt with 125 mg of hesperidin per 90 g serving. FY0 = frozen yogurt without added hesperidin.

The L* value indicates the lightness or darkness of a sample on a scale from 0 to 100 [46]. The treatment effect was significant (*p* < 0.05), but all the other effects were not significant (*p* > 0.05). The L* value decreased with increasing hesperidin concentration (Figure 4A). The darkest treatment was FY500, in which the highest hesperidin concentration was used, while the whitest frozen yogurt was FY0, containing no hesperidin. These results can be related to the results obtained by Binkowska [47], where this author established a decreasing L* value with increasing hesperidin concentration that was used in a hesperidin–silica complex. Several pigments, aside from white, might have an influence on the food color matrix by reducing the whiteness of the yogurt.

For the b* value (blueness–yellowness), the treatment effect and time effect were significant (*p* < 0.05). As expected, the b* value was higher in the treatments where the concentration of hesperidin was higher (Figure 4B). These results are similar to those found by Binkowska [47], where higher concentrations of hesperidin in a silica complex resulted in a higher b* value. This could be due to the yellowish-brown color of hesperidin [48]. After 30 days of storage, the b* value decreased slightly (Figure 4B), possibly due to the oxidation process during the storage of components such as riboflavin (vitamin B_2_) and β-carotene present in milk fat [49]. These results are very similar to those found by Kaur et al. [50], where they discovered a slight decrease of 2 units in the b* value for ice cream during four months of storage.

The a* values (redness–greenness) of the frozen yogurts with added hesperidin are shown in Figure 4C. All the effects were significant (*p* < 0.05). Increasing hesperidin concentrations slightly increased the obtained a* value (Figure 4C). These slight differences could be explained by the pigments present in hesperidin. The differences found over time and between treatments ranged from −0.47 to 0.14.

### 3.8. Bile Tolerance

**Figure 5:**
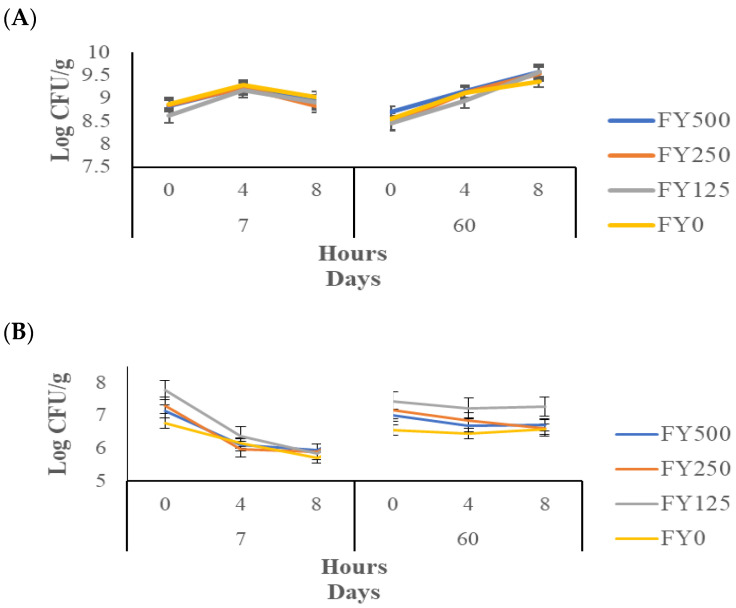

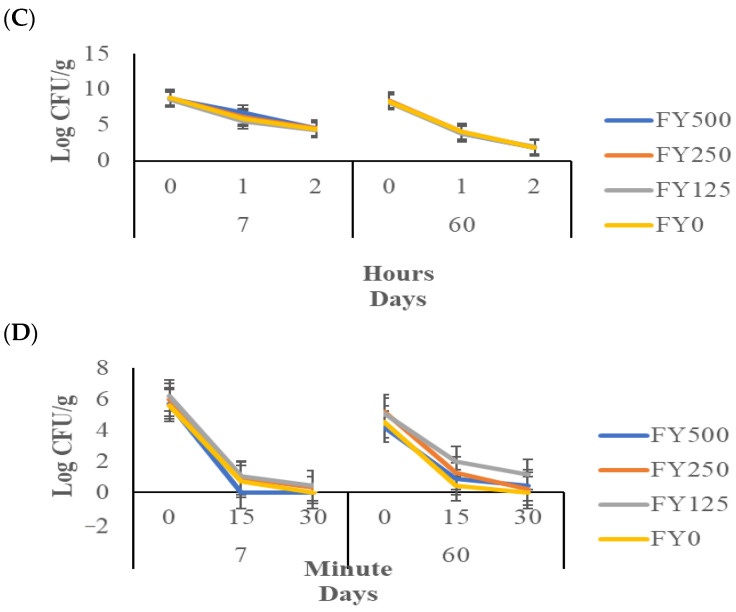
*S. thermophilus* (**A**) and *L. bulgaricus* (**B**) counts after exposure to bile salts (bile tolerance) and *S. thermophilus* (**C**) and *L. bulgaricus* (**D**) counts after exposure to acid (acid tolerance) in frozen yogurt with added hesperidin. FY500 = frozen yogurt with 500 mg of hesperidin per 90 g serving. FY250 = frozen yogurt with 250 mg of hesperidin per 90 g serving. FY125 = frozen yogurt with 125 mg of hesperidin per 90 g serving. FY0 = frozen yogurt without added hesperidin.

The bile tolerance results for *S. thermophilus* are shown in Figure 5A. This test measures the extent of the survival of bacteria exposed to bile salts [51]. The hour effect and the day*hour interaction effect were significant (*p* < 0.05), while the other effects were not significant (*p* > 0.05). After 60 d of storage and 8 h of incubation, the counts of *S. thermophilus* increased from 8.92 to 9.51 log cfu/g. These results suggest that cold stress improves bile tolerance after 60 d of storage. This could be due to the production of cold shock proteins (CSPs) by lactic acid bacteria during cold stress [52].

The bile tolerance of *S. thermophilus* has been investigated in other studies. Theegala et al. [53] added flaxseeds, which are rich in bioactive compounds, to an M17 broth with lactose but could not show an enhancement in the bile tolerance of *S. thermophilus* compared to the control without flaxseeds during 8 h of measurement in the presence of 0.3% bile salts (oxgall). Iyer et al. [54] tested strains of *S. thermophilus* for 3 h with 0.5, 1 and 2% of bile salts in pure broth, finding a consistent resistance of *S. thermophilus* to bile salts. *S. thermophilus* was shown to be a very stable bacteria throughout time, with a strong resistance to bile salts.

The results for the bile tolerance of *L. bulgaricus* are shown in Figure 5B. The hour effect, day effect and day × hour effect were significant (*p* < 0.05), while the remaining effects were not significant (*p* > 0.05). From d 7 to d 60, an increase was observed for the *L. bulgaricus* counts after 4 and 8 h of incubation.

Other studies have examined the bile tolerance of *L. bulgaricus*. The present results are different from those obtained by Vargas et al. [55], where they studied the influence of whey protein (1, 2 and 3%) on the bile tolerance of *L. bulgaricus*. In that study, it was found that the bacterial count during a 5 h period decreased by approximately 3 to 4 log cfu/g. These differences could be explained by the difference in the matrices in which the bacteria were tested since the experiment conducted by Vargas et al. [55] was performed in a pure broth, while the present experiment was performed in a yogurt matrix [56]. Additionally, Vargas et al. [55] highlighted the role of whey protein in the protection of *L. bulgaricus* since its survival rate increased. Moreover, Muramalla and Aryana [57] tested the bile tolerance of *L. bulgaricus* in skim milk at different homogenization pressures and found a reduction of less than 1 log cfu/g.

The present results demonstrate that *L. bulgaricus* and *S. thermophilus* can be considered good probiotic bacteria in terms of their bile resistance within a frozen yogurt matrix. This resistance against bile salts could be explained by the exopolysaccharides (EPSs) produced by lactic acid bacteria (LAB) during yogurt manufacturing [13].

### 3.9. Acid Tolerance

Acid tolerance can be defined as “the induced resistance to a normally lethal low pH challenge following growth or exposure at moderately low pH” [58]. Like bile tolerance, it is an important parameter for probiotic survival.

The results of the acid tolerance of *S. thermophilus* are shown in Figure 5C. The hour effect, day effect and day × hour effect were significant (*p* < 0.05), while the remaining effects were not significant (*p* > 0.05). A decrease was observed in the *S. thermophilus* counts after 1 and 2 h of incubation from 7 d to 60 d of storage. This could be caused by the prolonged stress of the freezing temperatures at which the bacteria were stored [59], making them more vulnerable to post-stress conditions. The results obtained in this study are similar to those found by Mena and Aryana [60], where they studied the influence of lactose on the acid tolerance of *S. thermophilus.* They demonstrated that after 120 min of incubation at a pH of 2.0, the bacterial count was reduced by 4 log cfu/g.

The acid tolerance of *L. bulgaricus* as influenced by hesperidin is shown in Figure 5D. The treatment effect, minute effect and day × hour effect were significant (*p* < 0.05), while the remaining effects were not significant (*p* > 0.05). The *L. bulgaricus* counts decreased as time progressed. When frozen yogurt was made by incorporating 250 mg of hesperidin per 90 g serving, the counts increased by less than 1 log cfu/g compared to the control samples. A low resistance of *L. bulgaricus* to adverse pH conditions was observed. Similar results were found by Boke et al. [56], where two out of four strains of *L. bulgaricus* tested at a pH of 2 did not survive (0%), and the other two strains survived at a rate of less than 50%. Even the buffer capacity of proteins found in the frozen yogurt was not sufficient to prevent the death of bacteria under these conditions [61]. 

### 3.10. Sensory Analysis and Purchase Intent of Frozen Yogurt

The results for the most relevant attributes (appearance, color, aroma, texture, iciness/sandiness, flavor, sourness and overall liking) are shown in Table 2. No statistical (*p* ˂ 0.05) differences were found among the treatments for appearance, color and aroma. On the other hand, the texture, iciness/sandiness, flavor, sourness and overall liking were significantly (*p* ˂ 0.05) different among the treatments. The highest concentration of hesperidin (FY500) influenced the consumers’ reactions to the flavor, sourness and overall liking by lowering the scores. These lower scores could have occurred because the flavonoids masked the sweet/sour taste with the bitterness of hesperidin [62,63]. The FY125 treatment and the control were not significantly (*p* ˂ 0.05) different in terms of the liking scores for any of the sensory attributes analyzed in this study.

Significant (*p* ˂ 0.05) differences for purchase intent were found (Table 3) when comparing whether the declaration of the health claim was made before or after asking about purchase intent for all the treatments containing hesperidin. As expected, health claims have a positive impact on consumers’ purchase intent [64]. In other words, incorporation of hesperidin had a positive impact in purchase intent of frozen yogurt [65].

## 4. Conclusions

This study showed that the addition of three different levels of hesperidin (125, 250 and 500 mg/90 g of product) did not influence the fermentation, overrun or viscosity of the frozen yogurt, meaning that no negative effect was perceived on its production. Hesperidin at these three concentrations did not significantly decrease the *S. thermophilus* and *L. bulgaricus* counts during the 60 days of storage. The highest hesperidin concentration significantly increased the hardness and decreased the melting rate. As expected, the L* and b* values were affected by this flavonoid changing its color properties. Nevertheless, the consumers did not notice a significant change in color in the sensory analysis. Moreover, the hesperidin addition did not negatively impact the survival rate of probiotic bacteria under adverse pH and bile salt conditions. 

The minimum concentration studied (125 mg/90 g of product) did not significantly affect any of the liking attributes for the sensory analysis compared to the control, which implies that this polyphenol could be used in commercial frozen yogurt without suffering an impact on its acceptability and likability. Also, the hesperidin addition positively impacted the purchase intent at all concentrations after a health claim was declared, implying that the incorporation of hesperidin seems to be an alternative for a market segment that demands healthier products. As a recommendation, it is suggested for future studies to evaluate other types of frozen desserts, such as ice cream or gelato, with other types of polyphenols, like resveratrol or quercetin. 

## Figures and Tables

**Table 1:** Formulations of frozen yogurts.

Compounds	Treatments			
	FY500	FY250	FY125	FY0
Milk (kg)	7.56	7.56	7.56	7.56
Sucrose (kg)	1.36	1.36	1.36	1.36
Nonfat dry milk (g)	399.52	399.52	399.52	399.52
Maltodextrin (g)	363.20	363.20	363.20	363.20
Corn starch (g)	20.00	20.00	20.00	20.00
*Lactobacillus* *bulgaricus* (g)	3.00	3.00	3.00	3.00
*Streptococcus* *thermophilus* (g)	3.00	3.00	3.00	3.00
Hesperidin (g)	59.66	29.74	14.85	-

FY500 = frozen yogurt with 500 mg of hesperidin per 90 g serving. FY250 = frozen yogurt with 250 mg of hesperidin per 90 g serving. FY125 = frozen yogurt with 125 mg of hesperidin per 90 g serving. FY0 = frozen yogurt without added hesperidin.

**Table 2:** Consumer liking score means and standard deviations for frozen yogurts with hesperidin added.

Attributes	FY500	FY250	FY125	FY0	
Appearance	6.37 ± 1.59 ^A^	6.74 ± 1.53 ^A^	6.50 ± 1.72 ^A^	6.72 ± 1.34 ^A^	
Color	6.43 ± 1.51 ^A^	6.69 ± 1.53 ^A^	6.67 ± 1.47 ^A^	6.74 ± 1.32 ^A^	
Aroma	5.79 ± 1.45 ^A^	5.87 ± 1.70 ^A^	5.57 ± 1.57 ^A^	5.66 ± 1.73 ^A^	
Texture	6.58 ± 1.66 ^AB^	6.25 ± 1.80 ^B^	6.84 ± 1.55 ^AB^	7.10 ± 1.58 ^A^	
Iciness/Sandiness	6.20 ± 1.87 ^AB^	6.11 ± 1.78 ^B^	6.41 ± 1.59 ^AB^	6.77 ± 1.69 ^A^	
Flavor	5.40 ± 2.23 ^B^	5.32 ± 2.23 ^B^	5.83 ± 2.23 ^AB^	6.43 ± 2.14 ^A^	
Sourness	4.99 ± 2.18 ^B^	5.10 ± 2.10 ^B^	5.48 ± 2.19 ^AB^	6.00 ± 2.06 ^A^	
Overall liking	5.66 ± 2.09 ^B^	5.42 ± 2.12 ^B^	5.96 ± 2.03 ^AB^	6.63 ± 1.88 ^A^	

FY500 = frozen yogurt with 500 mg of hesperidin per 90 g serving. FY250 = frozen yogurt with 250 mg of hesperidin per 90 g serving. FY125 = frozen yogurt with 125 mg of hesperidin per 90 g serving. FY0 = frozen yogurt without added hesperidin. ^A,B^ column means ± standard deviations not containing a common letter are significantly (*p* < 0.05) different.

**Table 3:** Distribution and probability (Pr > F) of purchase intent before and after declaring a health statement concerning health benefits provided by hesperidin addition.

Treatment	Purchase Intent before Declaring a Health Claim (%)		Purchase Intent after Declaring a Health Claim (%)		Pr > F
	Yes	No	Yes	No	
FY500	38.83	61.17	58.25	41.75	<0.0001
FY250	38.83	61.17	53.40	46.60	0.0007
FY125	50.49	49.51	65.05	34.95	0.0007
FY0	66.99	33.01	65.05	34.95	0.7905

FY500 = frozen yogurt with 500 mg of hesperidin per 90 g serving. FY250 = frozen yogurt with 250 mg of hesperidin per 90 g serving. FY125 = frozen yogurt with 125 mg of hesperidin per 90 g serving. FY0 = frozen yogurt without added hesperidin.

## Data Availability

The original contributions presented in the study are included in the article, further inquiries can be directed to the corresponding author.
